# Differential effects of overexpression of mutant huntingtin and TDP-43 in agouti-related protein neurons in the arcuate nucleus of the hypothalamus in mice

**DOI:** 10.1186/s40478-025-02201-x

**Published:** 2025-12-07

**Authors:** Jennifer Oraha, Ronja Wagner, Sofia Bergh, Nicola J Lee, Deniz Kirik, Åsa Petersén

**Affiliations:** 1 Translational Neuroendocrine Unit, Experimental Medical Science, Medical Faculty, Lund University, Lund, Sweden; 2 Charles Perkins Centre, University of Sydney, Sydney, NSW Australia; 3 School of Life and Environmental Sciences, Faculty of Science, University of Sydney, Sydney, NSW Australia; 4 Brain repair and Imaging in Neural Systems, Experimental Medical Sciences, Medical Faculty, Lund University, Lund, Sweden; 5 Department of Psychiatry, Skåne University Hospital, Lund, Sweden

**Keywords:** Arcuate nucleus, Agouti-related protein, Huntington disease, Frontotemporal dementia, Amyotrophic lateral sclerosis, Hypothalamus, Metabolism, Behavioural features, Neurodegeneration

## Abstract

The spectrum of frontotemporal dementia/amyotrophic lateral sclerosis (FTD/ALS) and Huntington disease (HD) are fatal neurodegenerative disorders with no major disease-modifying therapies. Recent work has shown that the hallmark pathological proteins TAR DNA binding protein of 43 kDa (TDP-43) in FTD/ALS and mutant huntingtin (mHTT) in HD may be interlinked. Furthermore, these disorders share early features of altered metabolism and psychiatric symptoms that have been suggested to arise from pathology in the hypothalamus, an important brain region involved in the regulation of metabolism and emotions. Agouti-related protein (AgRP)-expressing neurons localised exclusively to the arcuate nucleus (ARC) of the hypothalamus are key modulators of body weight regulation and food seeking behaviour, and they have recently been implicated in anxiety- and anhedonic-like processes. The aim of this study was to investigate the effects of overexpression of TDP-43 or mHTT in AgRP-expressing neurons on metabolic, behavioral and neuropathological features in mice. Flex-switch adeno associated viral vectors expressing human wild-type TDP-43, mHTT or green fluorescent protein to serve as a control, were injected into male and female AgRP-Cre mice to target the ARC using stereotactic surgery. We demonstrate targeted overexpression of transgenes including formation of mHTT inclusions in the ARC of the hypothalamus. Overexpression of mHTT led to a significant reduction in AgRP fibres in the hypothalamus 21 weeks post-injection, as well as higher food consumption in female mice. Overexpression of TDP-43 did not lead to the development of any metabolic or behavioral phenotypes in the mice. Our data suggest that AgRP neurons in the ARC are protected from the toxic effects resulting from overexpression of TDP-43 whereas they display some sensitivity to mHTT overexpression resulting in mHTT inclusion formation, reduction in AgRP fibers and sex-specific effects on food consumption. Taken together, other hypothalamic neuronal populations may be more important for the development of non-motor features resulting from overexpression of TDP-43 and mHTT in the hypothalamus.

**Supplementary Information:**

The online version contains supplementary material available at 10.1186/s40478-025-02201-x.

## Introduction

The spectrum of frontotemporal dementia (FTD)/amyotrophic lateral sclerosis (ALS) and Huntington’s disease (HD) are fatal neurodegenerative disorders clinically characterised by decline in cognitive and motor functions associated with pathology mainly originating in the cortical and striatal regions of the brain as well as spinal cord for FTD/ALS [1–4]. The nuclear-to cytoplasmic mislocalisation of TAR DNA binding protein of 43 kDa (TDP-43) in FTD/ALS and the formation of mutant huntingtin (mHTT) inclusions in HD are hallmarks of the pathology observed both in post-mortem cases and in different animal models of the disorders [5–11]. Interestingly, although the spectrum of FTD/ALS and HD have their hallmark pathological proteins, recent studies have shown TDP-43 aggregation in postmortem human brain tissue with HD, suggesting an overlap in the neuropathology of these diseases [12, 13]. These disorders are also characterized by early non-motor features including changes in body weight, appetite and psychiatric symptoms including apathy, depression and anxiety [14–20]. Interestingly, persons with HD and ALS often suffer from weight loss despite adequate caloric intake, whereas persons with FTD usually have increased appetite and an increased body mass index [16–20]. Elucidating the neurobiological pathways leading to the early metabolic and psychiatric features may shed light on potential novel targets for early therapeutic interventions in FTD/ALS and HD.

The hypothalamus is the master regulator of metabolism, emotional processing and sleep wake states. Several studies support the role of the hypothalamus as a key region for the metabolic and psychiatric features commonly observed in persons with FTD/ALS and HD [21–25]. Imaging and neuropathological studies on clinical cases with FTD/ALS and HD have shown early hypothalamic pathology with effects on neuronal populations involved in the regulation of metabolism, behavior and sleep [22, 23, 25–33]. We have recently shown that overexpression of TDP-43 in the hypothalamus using adeno associated viral (AAV) vectors led to similar neuropathology as observed in clinical cases with ALS including atrophy of the hypothalamus with loss of neuropeptide populations expressing orexin (hypocretin), oxytocin and melanin-concentrating hormone (MCH) [33, 34]. The mice also developed altered behavior as well as metabolic dysregulation, similar to the effects of overexpression of mHTT in the entire hypothalamus of mice [35–38].

The arcuate nucleus (ARC) of the hypothalamus is home to the orexigenic neuropeptide agouti-related protein (AgRP) [39–42]. AgRP neuronal cell bodies are located to the ARC but project fibres out to several hypothalamic sub-nuclei, including the lateral, ventromedial, paraventricular and dorsal hypothalamic areas [39, 43–47]. The innervation of these hypothalamic nuclei contributes to functions such as food intake, energy homeostasis and mood [44, 48, 49]. Recent work also suggests that AgRP neurons are activated by emotional stimuli and are involved in anxiety-like behaviours in mice [50–53].

Although the role of AgRP in food intake and body weight control is well established and more recently, a role in psychiatric-like behaviours has been suggested, the potential contribution of these neurons in the context of FTD/ALS and HD has not been well studied. The aim of this study was therefore to investigate the effects of overexpression of wild-type TDP-43 and mHTT in AgRP-expressing neurons on metabolism, behavior and neuropathology in mice.

## Materials and methods

### Animals

AgRP-Cre mice were obtained from the Jackson Laboratory (Strain number 012899) as AgRP-internal ribosome entry site (IRES)-Cre knock-in mice. Male and female AgRP-Cre mice were housed separately in cages of 2–5 animals and maintained in a 12 h light/dark cycle with *ad libitum* access to a standard chow diet, water and enrichment. The experimental procedures were approved by the Lund/Malmö Regional Ethical Committee, Sweden (Permit: 17113/2022).

### Validation of the AgRP-Cre mouse model

**Fig. 1:**
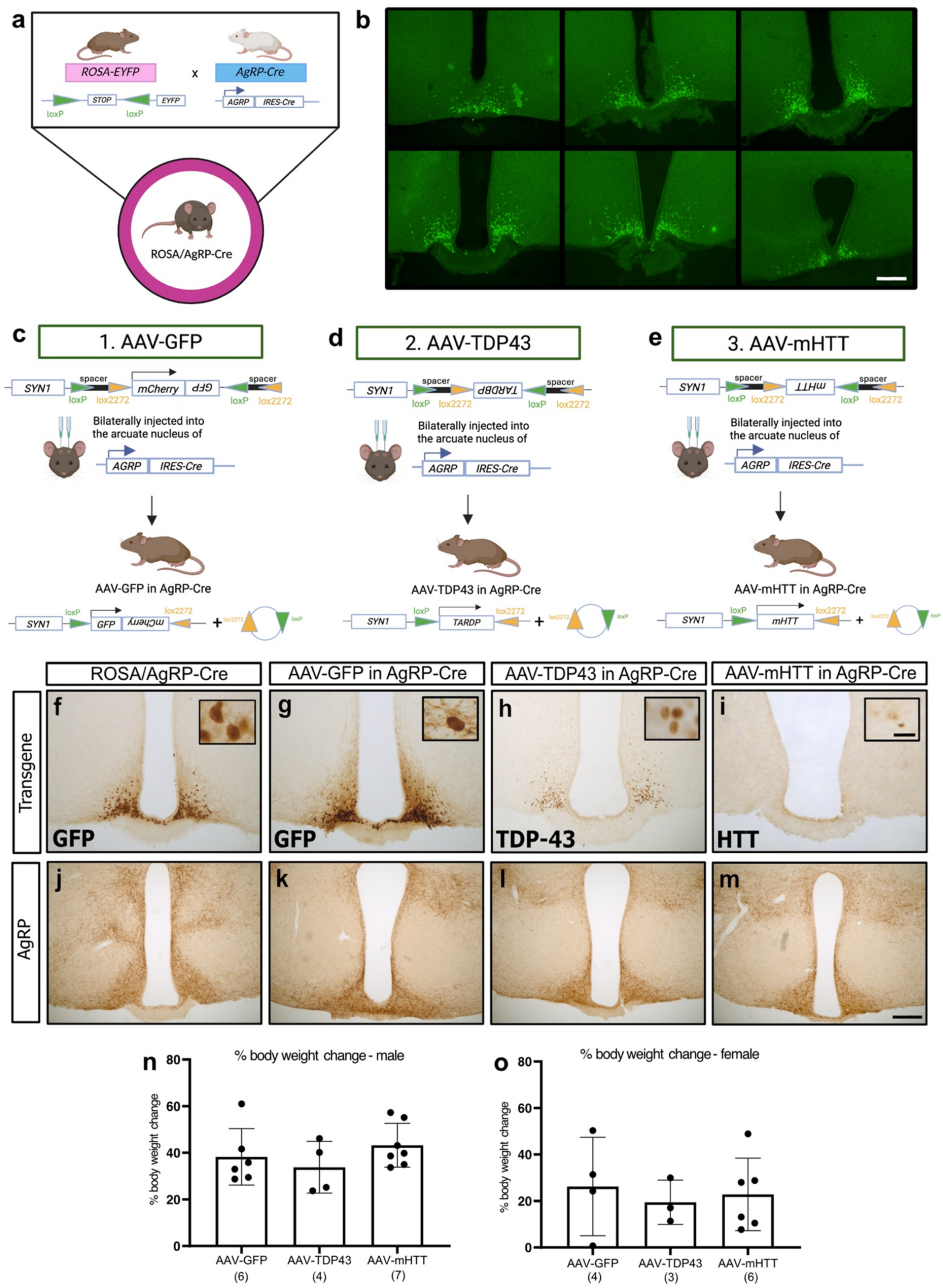
Overview of the experimental design. Schematic of the cross-breeding of the reporter line ROSA-EYFP with AgRP-Cre mice resulted in ROSA/AgRP-Cre mice. The stop codon upstream of the EYFP gene is flanked by two loxP sites, as depicted by green triangles, which allows yellow fluorescent protein (EYFP) to be expressed exclusively in AgRP-expressing neurons in the presence of Cre (**a**). Representative images of brain sections cut at 30 μm in 6 series from ROSA/AgRP-Cre mice ranging from bregma − 1.12 mm to −2.70 mm show targeted expression of green fluorescent protein (GFP) in AgRP-expression neurons in the arcuate nucleus (ARC, **b**). Schematic representation of flex-switch adeno-associated viral (AAV) vector construction and effects after stereotactic injections in AgRP-cre mice (**c**–**e**). The three vectors express either GFP, TDP-43 or mHTT under the human synapsin-1 (SYN1) promoter, flanked by two Lox sites on both sides of the gene of interest: LoxP in green and Lox2272 in orange. The two Lox sites on either end of the gene of interest are separated by a spacer of 24-base pairs with a non-coding sequence to prevent off-target effects. In the presence of Cre, Lox sites of the same type can recombine, resulting in inversion of the gene of interest. Representative images illustrating GFP in the ARC of ROSA/AgRP mice (**f**) as well as GFP (**g**), TDP-43 (**h**) and mHTT (**i**) in AgRP-Cre mice at 10-weeks post injection. Immunohistochemistry for AgRP shows AgRP fibre expression in the ARC and the hypothalamus with no apparent effects on the intensity or pattern of AgRP-immunopositivity of TDP-43 or mHTT overexpression in AgRP-expressing neurons compared to ROSA/AgRP-Cre and GFP overexpression in the ARC (**j**–**m**). No difference in percentage of body weight gained between groups, regardless of sex and vector at 10-weeks post-injection (**n**–**o**). Data are presented as mean ± standard deviation (SD). Data was analyzed by two-way ANOVA for the influence of sex and vector, with a subsequent one-way ANOVA if there was effect of sex or vector. The number of animals in each group is indicated in parentheses. Scale bar in b, m = 200 μm. Scale bar in i insert = 5 μm

In the AgRP-Cre mouse model, the IRES-Cre recombinase (Cre) sequence is inserted downstream of the stop codon in AgRP locus, ensuring that Cre is specifically expressed in neurons that express AgRP. In order to validate this animal model, AgRP-Cre mice were crossed with the B6.129 × 1-Gt(ROSA)26-Sortm1(EYFP)Cos/J (ROSA-EYFP) reporter mice (Jackson Laboratories) which carries the enhanced yellow fluorescent protein (EYFP) gene downstream of a stop codon flanked by two loxP sites, resulting in a crossbreed referred to as ROSA/AgRP-Cre (*n* = 5; n_male_=2, n_female_=3). The ROSA/AgRP-Cre crossbreed achieves the expression of EYFP specifically in AgRP expressing cells, due to the excision of the stop codon by Cre positioned upstream of the *EYFP* gene sequence (Fig. 1a).

### Flex-switch adeno-associated viral vectors

Flex-switch AAV2/5 vectors were produced using a method of double transfection as described previously [54, 55]. The transgene of interest was flanked by inverted terminal repeats (ITR) from AAV2 and packaged in a capsid from AAV5. In a flex-switch system, in the presence of Cre, Lox sites of the same type can recombine, resulting in inversion of the gene of interest. This results in either expression (if the transgene is in an antisense orientation) or silencing (if the transgene is in a sense orientation). The gene of interest was flanked by two lox sites, LoxP and Lox2272 on both sides. These lox sites on either end of the gene of interest were separated with the use of a 24-base-pair spacer to prevent off target effects. The sequence of the spacer was as follows: AGTGCCATAGTGCCATAGTGCCAT.

Two flex-switch AAV vectors were designed expressing human wild-type TDP-43 (AAV-TDP43) or the first 909 amino acids of the N-terminal fragment of the human *HTT* gene with 78 polyglutamines (AAV-mHTT). The transgene sequences were regulated under the human synapsin-1 promoter. A polyadenylation tail was integrated to increase the mRNA half-life by protecting the transcript against endogenous endonuclease activity. A similar flex-switch vector expressing green fluorescent protein (GFP) as described previously was also used (AAV-GFP) as a control [55]. Titers were estimated by TaqMan quantitative polymerase-chain reaction.

### Quantitative polymerase chain reaction of flex-switch AAV-vectors

rAAV titers were determined by quantitative polymerase chain reaction (qPCR) using primers and hydrolysis probe targeting the ITR sequence [56]. rAAV samples were lysed using SDS. The primers and probe were synthesised by Eurofins Genomics. The plasmid pTRUF20, linearized outside the rAAV2 genome using the HindIII restriction enzyme (ThermoFisher Scientific, Catalog #FD05004), was used to generate a standard curve for the qPCR. The concentration of isolated plasmid DNA was determined through 260 nm absorbance measurements using a Nanodrop 2000 spectrophotometer (ThermoFisher Scientific), according to the manufacturer’s instructions. The qPCR was done using a LightCycler 480 instrument II (Roche) and LightCycler 480 Probes Master Reagents (Roche, Catalog #04707494001), according to the manufacturer’s instructions. The qPCR measurements were analyzed for titer determination using the LightCycler 480 Software version 1.5.1.62. The titer determination was made to ensure that all vectors were around 2.0 × 10^14^ genome copies(gc)/ml after dilution of the stock vectors. The exact titers for the AAV vectors used for the first group of mice were 2.2 × 10^14^ gc/ml for AAV-GFP, 2.0 × 10^14^ gc/ml for AAV-TDP43 and AAV-mHTT. For the second group, the titers were 2.1 × 10^14^ gc/ml for AAV-GFP and AAV-TDP43 and 2.2 × 10^14^ gc/ml for AAV-mHTT.

### Stereotactic injections of flex switch AAV vectors

At 8 weeks of age, male and female mice AgRP-Cre were anaesthetised with a mixture of 2% isoflurane and N_2_O and transferred onto a stereotactic instrument. Briefly, the animal’s skull was thinned with a dental drill at the chosen location, and the thin bone flap was carefully removed, leaving the dura intact. Flex-switch AAV vectors were bilaterally injected into AgRP-Cre mice using the following coordinates, with bregma as the origin to target the ARC of the hypothalamus: 0.7 mm posterior to the bregma, ± 0.55 mm lateral to the bregma and 5.2 mm ventral to the dura. The bilateral injections were performed using a 5 µl Hamilton (Nevada, USA) syringe attached to a pulled glass capillary with an outer diameter of 80 μm. A total of 0.5 µl viral vector solution was injected at the site of the coordinates at a rate of 0.05 µl/15s with a concentration of 2.0-2.2 × 10^14^ gc/ml for all vectors. The needle was left in place for an additional five minutes in situ after infusion of the viral vector before being slowly retracted. Following the procedure, the skull was cleaned, and the incision was closed with metal clips. Mice were placed onto a heating mat and monitored until they gained consciousness again before being returned into home cages.

## Experimental design

**Fig. 2:**
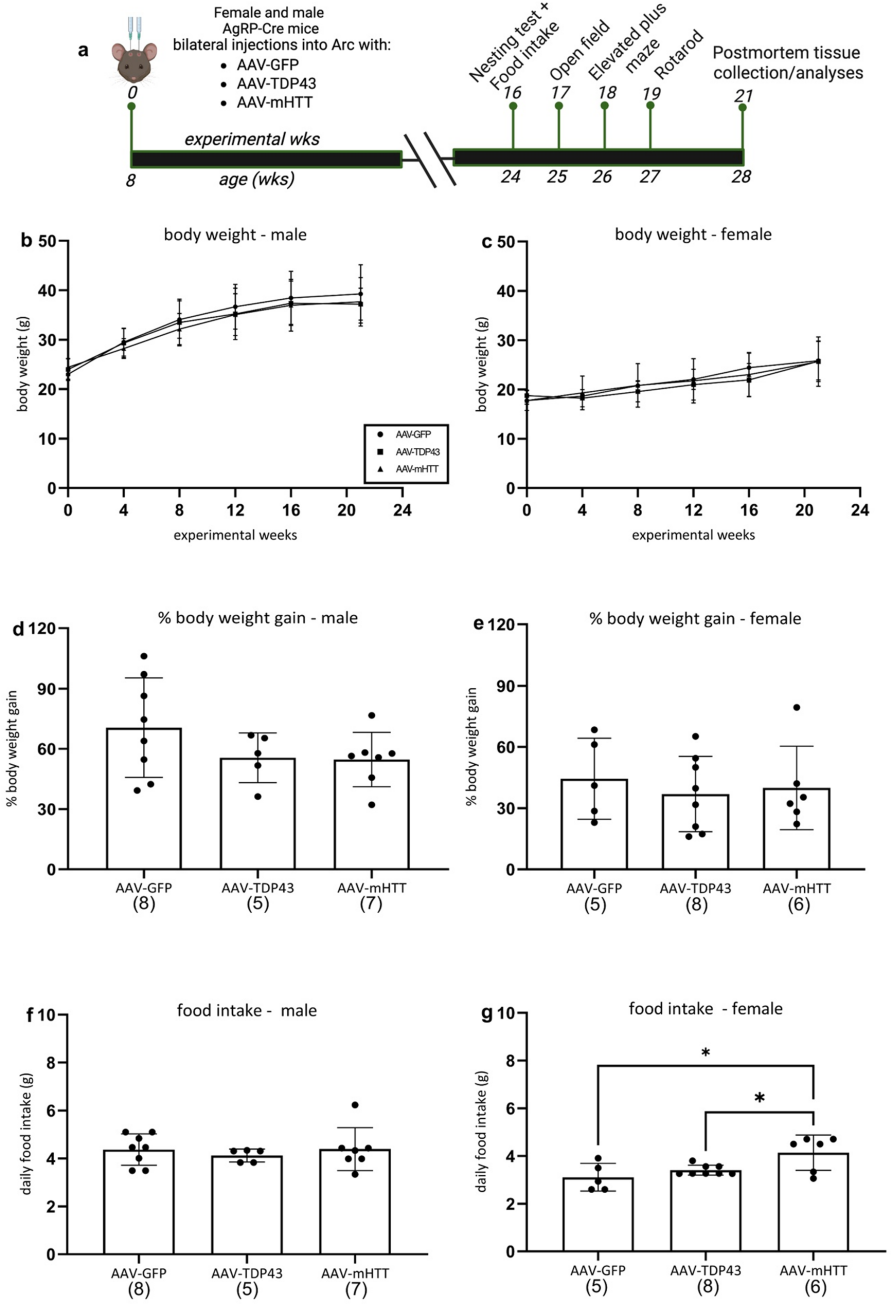
Assessment of body weight and food intake after overexpression of TDP-43 and mHTT in AgRP-Cre mice using flex-switch AAV vectors. Experimental timeline of AgRP-Cre mice injected with AAV-GFP, AAV-TDP43 or AAV-mHTT including metabolic and behavioural analyses conducted starting from post-injection week 16 to 19, with an endpoint at 21-weeks post-injection (**a**). No effect on body weight over the examined 21 weeks period post-injection in male or female mice (**b**–**e**). Food intake was assessed 16-weeks post injection over a 4 day period, with measurements in grams made at day 0 and day 4 and divided by number of animals per cage (*n* = 2 or 3, with the exception of 2 animals single housed) and days (*n* = 4). No effect on daily food intake in male mice (**f**), however food intake was significantly increased following mHTT overexpression in female mice compared to GFP (g, *=0.013) and TDP-43 (*=0.049). Data are presented as mean ± standard deviation (SD). Data was analyzed by two-way ANOVA for the influence of sex and vector group, with a subsequent one-way ANOVA if the effect of sex or vector group was established, followed by Tukey’s multiple-comparison test of significant effects. The number of animals in each group is indicated in parentheses

To validate the selective targeting of AgRP neurons in the ARC and investigate potential neuropathological effects, we first injected flex-switch AAV vectors into AgRP-Cre mice at 8 weeks of age and sacrificed them 10 weeks post-injection (AAV-GFP (*n* = 10; n_male_=6, n_female_=4), AAV-TDP43 (*n* = 7; n_male_ = 4, n_female_=3) and AAV-mHTT (*n* = 13; n_male_=7, n_female_=6)). To further investigate the neuropathological effects as well as effects on metabolism and behaviour, we also injected flex-switch AAV vectors into the ARC of another group of AgRP-cre mice at 8 weeks of age and sacrificed them at a later time-point, i.e. at 21 weeks post-injection (AAV-GFP (*n* = 13; n_male_=8, n_female_=5), AAV-TDP43 (*n* = 13; n_male_=5, n_female_=8 ) and AAV-mHTT (*n* = 13; n_male_=5, n_female_= 8)). Body weight measurements were made every four weeks for both groups of mice, and a battery of metabolic and behavioural tests were conducted starting at 16-weeks post-injection until sacrifice at 21-weeks post-injection for the second group of mice. Each behavioural test was performed by the same experimenter, with one week between each test (Fig. 2a).

### Nesting test and food intake

Nesting and food intake were assessed 16-weeks post-injection over a 4-day period (with measurements for nesting ability taken at day 2 and 4 and food intake from day 0 and 4) as previously described [34]. Nest building is an innate behaviour in laboratory mice and can serve as an early indicator of several behavioural deficits such as cognitive decline, anxiety and apathy [57]. Mice injected with the same vector type were housed in pairs or groups of three, with no single housing permitted according to the ethical permit – with the exception of two mice single housed due to aggressive behaviour. Following a 2-day acclimation period, at day 0 of the nesting behaviour analysis, each cage was provided with 7–8 g of intact pre-patterned nesting material. Nesting ability was assessed on experimental day 2 and 4 based on a modified scoring system from Gaskill et al 2013 and as previously described in Bergh et al., 2025 [34, 68]. To assess nesting ability, the scoring system was designed to rate the level of manipulation of the nesting material according to the following criteria: a score of 0 was assigned if the material appeared untouched, a score of 1 was given for manipulated material without starting to cup or form a dome, a score of 2 indicated manipulated material and a partially formed nest with a developing cup and a score of 3 was given for complete manipulation of material with a fully cupped dome. For evaluation of nesting, the experimenter was blinded to the experimental groups. Additionally, daily food intake was measured over four days by calculating the difference in total food weight between day 0 and day 4 and divided by the number of animals per cage (2 or 3) and number of experimental days (4).

### Open field test (OF)

General locomotor activity was assessed 17-weeks post-injection. Mice were individually placed in the centre of a 40 × 40 × 40.5 cm arena and given 60 min to freely explore. Each station was cleaned with 70% ethanol between each test. The recording and analysis to calculate the total activity was done using the Ethovision XT 13 software system (Version 13, Noldus Information Technology, Wageningen, Netherlands). Mice were moved into the testing area 1 h before the test and were assessed in a randomized fashion. All experiments were performed between 8:00–13:00.

### Elevated plus maze (EPM)

Anxiety-like behaviour was assessed 17-weeks post-injection using the Elevated plus maze (EPM) as previously described by Pellow [58]. The maze was elevated 55 cm above its base, consisting of two closed arms and two opposing open arms (each arm measuring 35 × 7.5 cm), with an open centre connecting all four arms (7.5 × 7.5 cm). The walls of the closed arms had a height of 30 cm. In brief, each mouse was placed in the centre of the apparatus and given 5 min to freely explore. During this time, the mouse was recorded using the Ethovision XT 13 software system in order to assess the total distance moved as well as the percentage of time spent in the open versus closed arms. The EPM apparatus was cleaned with 70% ethanol between each test. Analysis was performed using the Ethovision XT 13 software system. Mice were moved into the testing area 1 h before the test and were assessed in a randomized fashion. All experiments were performed between 8:00–13:00.

### Accelerating Rotarod test (RR)

Motor coordination and balance were assessed at 18-weeks post-injection with a rotarod test using the Rotamex 4/8 System (Rota Rod Columbus Instruments, US). The rod diameter was 3 cm, and the fall height was 20 cm. Vertical barriers were used to separate individual mice during the test. Prior to the testing day, mice were trained for three rounds for three consecutive days at a fixed speed of 5 rotations per minute (rpm) for 2 min. On the fourth day, mice were tested three times with linearly accelerating speeds (5–40 rpm over 5 min) separated by 2 h rest between each test. Mean latency to fall over the three tests was used as a measure of rotarod performance. The rotarod apparatus was cleaned with 70% ethanol between each test. Mice were moved into the testing area 1 h before the test and were assessed in a randomized fashion. All experiments were performed between 8:00–13:00.

### Immunohistochemistry

#### Peroxidase-based immunohistochemistry

Mice were sacrificed using sodium pentobarbital (150 mg/kg i.p.) and transcardially perfused with saline (0.9%) and then pre-cooled 4% paraformaldehyde (PFA) in 0.9% saline solution. Brains were post-fixed in PFA for 24 h and then placed in 25% sucrose for cryoprotection. Brains were sectioned frozen in the coronal plane at a thickness of 30 µm in six series. Peroxidase-based immunohistochemistry was carried out as described previously [55]. One series of free-floating sections was processed using primary antibodies raised against GFP (1:20,000, raised in rabbit, ab13970, Abcam), TDP-43 (1:100,000, raised in rabbit, 10782-2-AP, Proteintech), HTT (1:500, raised in goat, sc-8767, Santa-Cruz), AgRP (1:10,000, raised in rabbit, H-003-57, Phoenix Pharmaceuticals), ubiquitin (1:2,000, raised in rabbit, Z0458, DakoCytomation), ionized calcium-binding adapter molecule 1 (Iba1, 1:1000, raised in rabbit, 019-19741, FUJIFILM Wako Pure Chemical Corporation), and Sequestesome 1 (p62, 1:5,000, raised in Guinea Pig, GP62-C, Progen). Sections were labelled with a suitable biotinylated secondary antibody (1:200, anti-rabbit, anti-goat or anti-guinea pig, Vector Laboratories) and visualised with 3,3’-diaminobenzidine (DAB) as chromogen using hydrogen peroxidase. The sections were then mounted onto chromalum gelatinized slides, passed through a series of ethanol and xylene baths for dilapidation and cover-slipped with DPX mounting media (Sigma) and processed for microscopy the following day. In addition, sections from three mice injected with AAV-mHTT already immunohistochemically processed for HTT and ubiquitin, were counterstained for cresyl violet (CV) for 60–90 s respectively according to the manufacturer’s instructions (Cresyl Violet Acecate, Sigma-Aldrich, C5042-10G) before being rinsed in water, dehydrated and re-coverslipped with DPX.

#### Immunofluorescence

For analyses of intracellular TDP-43 localization, AAV-TDP43 injected AgRP-Cre brain sections at 21-weeks post injection were processed for immunofluorescence as described previously [55]. One series of free-floating sections was incubated with a primary antibody against TDP-43 (1:1000, raised in rabbit, 10782-2-AP, Proteintech) overnight followed by an incubation with Alexa Fluor 647-conjugated secondary antibody (1:200, Jackson Laboratories) for 2 h. After secondary antibody incubation, samples were stained with Hoechst 33,343 (1:1000, 62249, Thermo Fisher) for 5 min. All sections were then mounted onto chromatin-gelatin coated glass slides and coverslipped using VectaShield mounting medium (Vector Laboratories) and stored in the cold room until image acquisition.

#### Image acquisition

Brightfield and fluorescence image acquisition was performed using the Zeiss Axio Imager M2 equipped with Axiocam 305 colour 5-megapixel camera for imaging of brightfield and Axiocam 807 mono 7-megapixel camera for imaging of fluorescent signals. Images in this study were acquired with apochromatic objectives (10x/0.3 NA, 20x/0.8 NA and 40x/1.3 NA oil). All images were acquired using consistent exposure and contrast settings. Post-processing was carried out using the Zeiss Zen 3.8 software. Schematics were creating using BioRender and image panels were created and edited using Affinity Designer 2.0.

#### Intensity quantification

Brains collected at 21-weeks post injection were sectioned and processed immunohistochemically for AgRP and Iba1. All sections were imaged on a Zeiss Primostar 3 microscope using a 10 × (0.3 NA) objective. For quantification, the ImageJ 1.5t software was used by converting all images to 8-bit and then setting a constant threshold for AgRP immunopositive fibres (0-120, 3.73%) and for Iba1 (0-110, 3.4%). The threshold value represents pixels within the ROI range and percentage value represents the percentage of pixels in the ROI that are included in the threshold. The ARC and entire hypothalamus were identified bilaterally at bregma levels between − 1.70 and − 1.94 mm according to the Franklin and Paxinos mouse brain atlas (3rd Edition) and a region of interest (ROI) was delineated for ARC and the whole hypothalamus. For AgRP in ARC, AgRP in hypothalamus and Iba1 in ARC, the mean gray value was measured.

#### Quantification of number of cells with cytoplasmic TDP-43 translocalization

Brains collected at 21-weeks post injection were sectioned and processed for fluorescent immunohistochemistry for TDP-43 (*n* = 3). Images were acquired using the Zeiss Axio Imager M2 equipped with the Axiocam 807 mono 7-megapixel camera for imaging of fluorescent signals. In order to quantify the number of cells with cytosolic localization of TDP-43, a Z-stack of three sections per mouse was taken at 20x magnification and processed through ImageJ 1.5T software. Briefly, images were uploaded to ImageJ, Z-stacks were compiled to achieve one single image with all planes visible. For estimation of number of cells with cytoplasmic TDP-43 immunopositivity, 76 to 99 TDP-43 overexpressing cells per mouse were counted manually using the Multipoint tool in ImageJ.

### Statistical analyses

The statistical analyses were conducted using IBM SPSS statistics software (Version 29.0.2.0). All data were first assessed for normal distribution. If the data were not normally distributed, log10 or square root transformation was applied to achieve normal distribution, enabling the use of parametric tests. Data were analysed either by two-way analyses of variance (ANOVA) followed by one way ANOVA and Tukey’s multiple-comparison test or an independent samples-Kruskal-Wallis test. Significant statistical difference was accepted at *p <* 0.05, and the data are expressed as mean, mean ± standard deviation (SD) or median ± range. A full report of the statistical analyses is presented in Appendix S1.

## Results

### Validation of the AgRP-Cre mouse model and flex-switch AAV vectors overexpressing GFP, TDP-43 and mHTT in AgRP-Cre mice

To validate the AgRP-Cre model, we crossbred AgRP-Cre mice with ROSA-EYGP reporter mice (Fig. 1a). In these mice (*n* = 5), we observed EYFP-expressing cells confined to the region corresponding to the ARC, indirectly confirming a correct targeted expression of EYFP in AgRP neurons (Fig. 1b). We then injected AAV vectors expressing GFP (*n* = 10), TDP-43 (*n* = 7) or mHTT (*n* = 13) bilaterally into the ARC of male and female AgRP-Cre mice (Fig. 1c-e). We compared the AAV vector injected mice to ROSA/AgRP-Cre as a reference for the localization of AgRP neurons in the ARC of the hypothalamus (*n* = 5). We observed overexpression of TDP-43 in the ARC (Fig. 1h), as well as targeting of ARC neurons with the AAV-GFP vector serving as a control vector (Fig. 1g). Additionally, at 10-weeks post injection, we observed a few cells within the ARC displaying mHTT- immunopositive inclusions, indicating that AgRP neurons have the ability to form mHTT inclusions (Fig. 1i).

### Effects on AgRP fibers and body weight gain 10 weeks post-injection of flex-switch AAV-vectors expressing TDP-43 or mHTT in the ARC

In order to determine whether overexpression of TDP-43 or mHTT in the ARC would affect AgRP-expressing neurons, we conducted immunohistochemistry using an antibody against AgRP (Fig. 1j–m). Antibodies against AgRP typically target the fibers and not cell bodies [59–61]. In line with previous studies on this neuronal population, we found that the AgRP antibody stained fibers. Upon qualitative assessment, AgRP immunoreactivity did not appear altered between any of the vector groups at 10-weeks post-injection (Fig. 1j–m). Additionally, a two-way ANOVA analyses indicated a sex-dependent effect *(p* = 0.006) on body weight at 10-weeks post-injection; therefore, the data were further analysed according to sex (Fig. 1n, o). However, a one-way ANOVA revealed no differences in males or females in percentage of body weight gained at this time point.

### Overexpression of mHTT but not TDP-43 leads to increased food intake, while body weight remains unchanged

AgRP neurons are well known to regulate energy balance and food seeking behaviour [49]. We therefore evaluated body weight every 4 weeks until 21 weeks post-injection, the percentage of body weight gain at 21 weeks post-injection as well as food intake in AgRP-Cre mice injected with AAV-TDP43 or AAV-mHTT. The body weight curves were similar for the three groups (Fig. 2b, c). A two factor ANOVA revealed a significant effect of sex on body weight gain at 21-weeks post injection (*p* = 0.04), therefore the body weight data are presented and further analyzed separately according to sex. One-way ANOVA revealed no significant difference in the percentage of body weight gained in either males or female following TDP-43 or mHTT overexpression (Fig. 2d, e).

Additionally, daily food intake was measured in grams across a 4-day period, with food being measured on day 0 and compared to the measurement at day 4. Total food intake across the 4-day period was then divided by the number of mice per cage (2 or 3, except for 2 animals who were single housed) and then divided by the number of days (4) (Fig. 2f, g). A two factor ANOVA revealed a significant effect on sex (*p* < 0.001) therefore the food intake data are presented separately according to sex. A one- way ANOVA revealed AAV-mHTT injected females had a higher food intake per day compared to AAV-GFP (Fig. 2g, *p* = 0.013) and AAV-TDP43 injected females (*p =* 0.049). There were no differences between the groups in males at this time point (Fig. 2f).

### Overexpression of TDP-43 or mHTT in AgRP neurons has no effect on locomotor activity

**Fig. 3:**
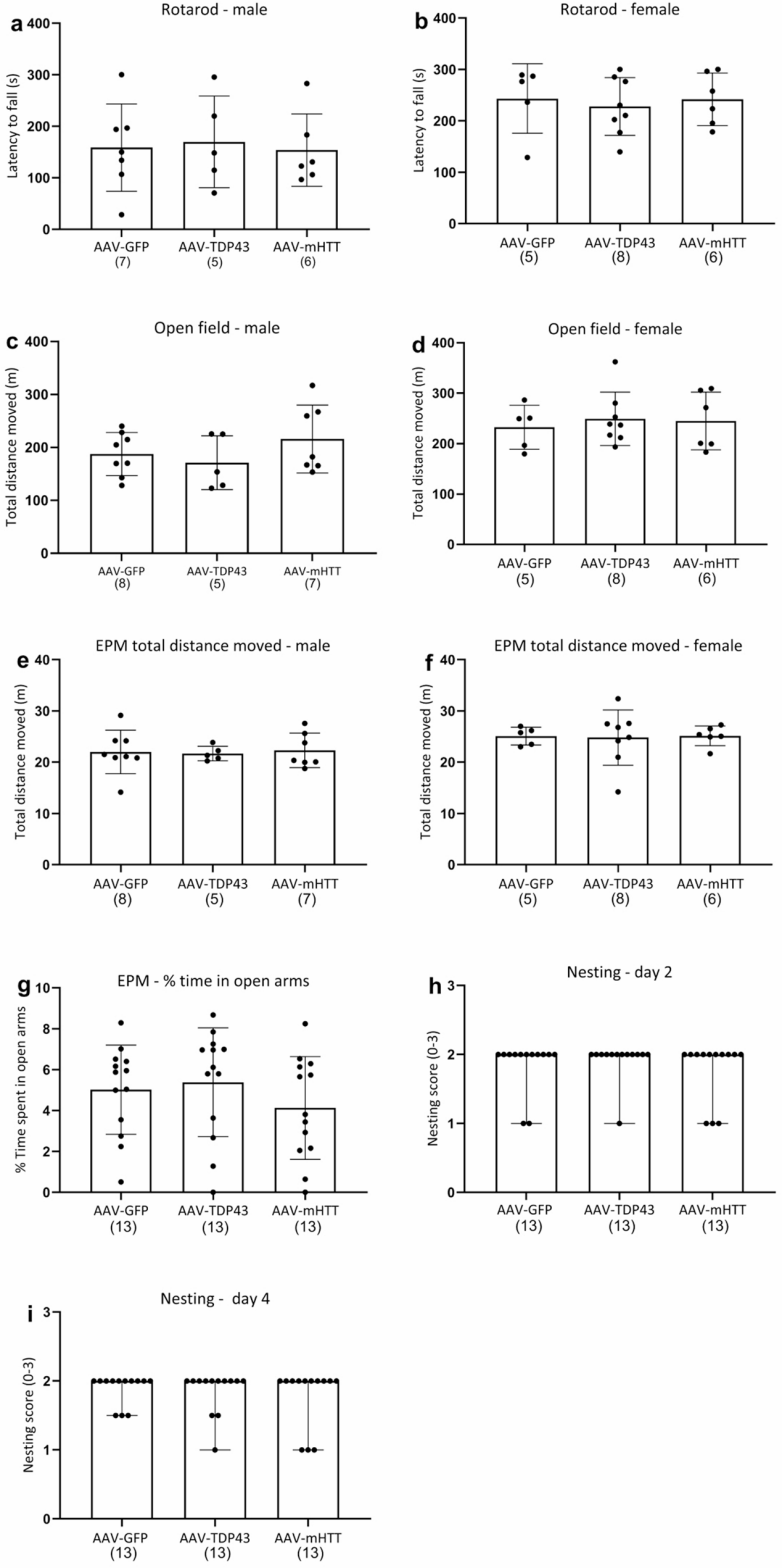
Behavioural phenotyping of AgRP-cre mice injected with AAV-TDP43, AAV-mHTT and AAV-GFP. There was no difference in latency to fall in the rotarod test at 18-weeks post-injection between groups, regardless of sex or treatment (**a**–**b**). There was no difference in distance travelled in the open field at 17-weeks post injection between groups, regardless of sex or treatment (c-d). No differences in total distance moved between groups were detected in the Elevated plus maze (EPM) at 17-weeks post injection, regardless of sex or vector (**e**,** f**). There were no effects on anxiety-like behavior as assessed as percentage spent in open arms at 17-weeks post injection (**g**). Assessment of nest building at 16-weeks post injection on day 2 (**h**) and 4 (**i**) revealed no significant difference across the groups, regardless of sex or vector. Data are presented as mean ± standard deviation (SD) (**a**–**g**) or median ± range (**h**–**i**) Data was either analysed by two-way ANOVA for the influence of sex and vector, with a subsequent one-way ANOVA if the effect of sex or vector group was established (**a**–**g**) or independent samples-Kruskal-Wallis test (**h**–**i**) for the influence of sex and vector. The number of animals in each group is indicated in parentheses (NB: n_males_ (a) = 7. One male was euthanised due to illness as per our ethical protocol)

As motor dysfunction is part of TDP-43 proteinopathies and HD, we investigated the effect of overexpression of TDP-43 and mHTT in AgRP neurons on locomotor activity. By employing the OF and RR tests, we assessed distance travelled and latency to fall (Fig. 3a–d). Analysis using two-way ANOVA revealed a significant effect of sex on latency to fall in RR (Fig. 3a–b, *p* = 0.003), therefore the data is presented according to sex. However, one-way ANOVA revealed no significant differences between the groups for either males or females in latency to fall at 18-weeks post-injection. Similarly, analysis using two-way ANOVA revealed a significant effect of sex (Fig. 3c–d, *p* < 0.001) on the total distance moved in OF, therefore the data is presented according to sex. However, one-way ANOVA revealed no significant differences between the groups for either males or females in the total distance moved. Hence, overexpression of TDP-43 and mHTT in the ARC did not affect motor activity in mice at the time-point analyzed in this study.

### Overexpression of TDP-43 or mHTT in the ARC of AgRP-Cre mice does not affect anxiety -like behaviour

Anxiety is part of the psychiatric features in persons with HD and FTD/ALS. As a means to investigate anxiety-like behaviour in mice, we employed the EPM at 17-weeks post-injection in AgRP-Cre mice. Two-way ANOVA revealed an effect on sex (*p =* 0.016) in total distance moved, and the data was therefore presented according to sex (Fig. 3e–f). One-way ANOVA showed no difference in total distance moved between the groups, similar to the results shown in our motor tests. There was no effect on sex (*p* = 0.718) or vector (*p =* 0.502) on the percentage of time spent in open arms, a parameter indicative of anxiety-like behaviour, and the data was therefore collapsed across sex (Fig. 3g). These results indicate no major alterations in anxiety-like behaviour in male or female AgRP-Cre mice injected with AAV-TDP43 or AAV-mHTT compared to AgRP-Cre mice injected with AAV-GFP.

### Overexpression of TDP-43 or mHTT in AgRP neurons has no effect on nesting behaviour

Given the involvement of AgRP neurons in social behaviour, cognition and anhedonic behaviours [62–64] we sought to investigate the effects of TDP-43 and mHTT overexpression on apathy-like behaviour by evaluating nest construction in this experiment. Deviations in nest-building activity, such as the inability to construct a well-structured nest, may be an indicator of apathy-like behaviour, a common feature in individuals with FTD/ALS and HD [65–67]. Nest construction was analysed with our scoring system as described in Bergh et al., 2025, adapted from Gaskill et al., 2013 [34, 68]. Nest construction was assessed on experimental days 2 and 4. A two-way ANOVA revealed no sex-dependent effect on either day 2 (*p* = 0.180) or day 4 (*p* = 0.276), therefore the data were collapsed across sex (Fig. 3h–i). There were also no differences between the vectors on day 2 (*p =* 0.689) nor day 4 (*p* = 0.209). This data shows that overexpression of TDP-43 or mHTT in the ARC of AgRP-Cre mice does not affect nest-.

building behaviour at the time-point examined.

### Reduction of AgRP fibres in the entire hypothalamus following mHTT but not TDP-43 overexpression, while AgRP fibres within the ARC are spared

**Fig. 4:**
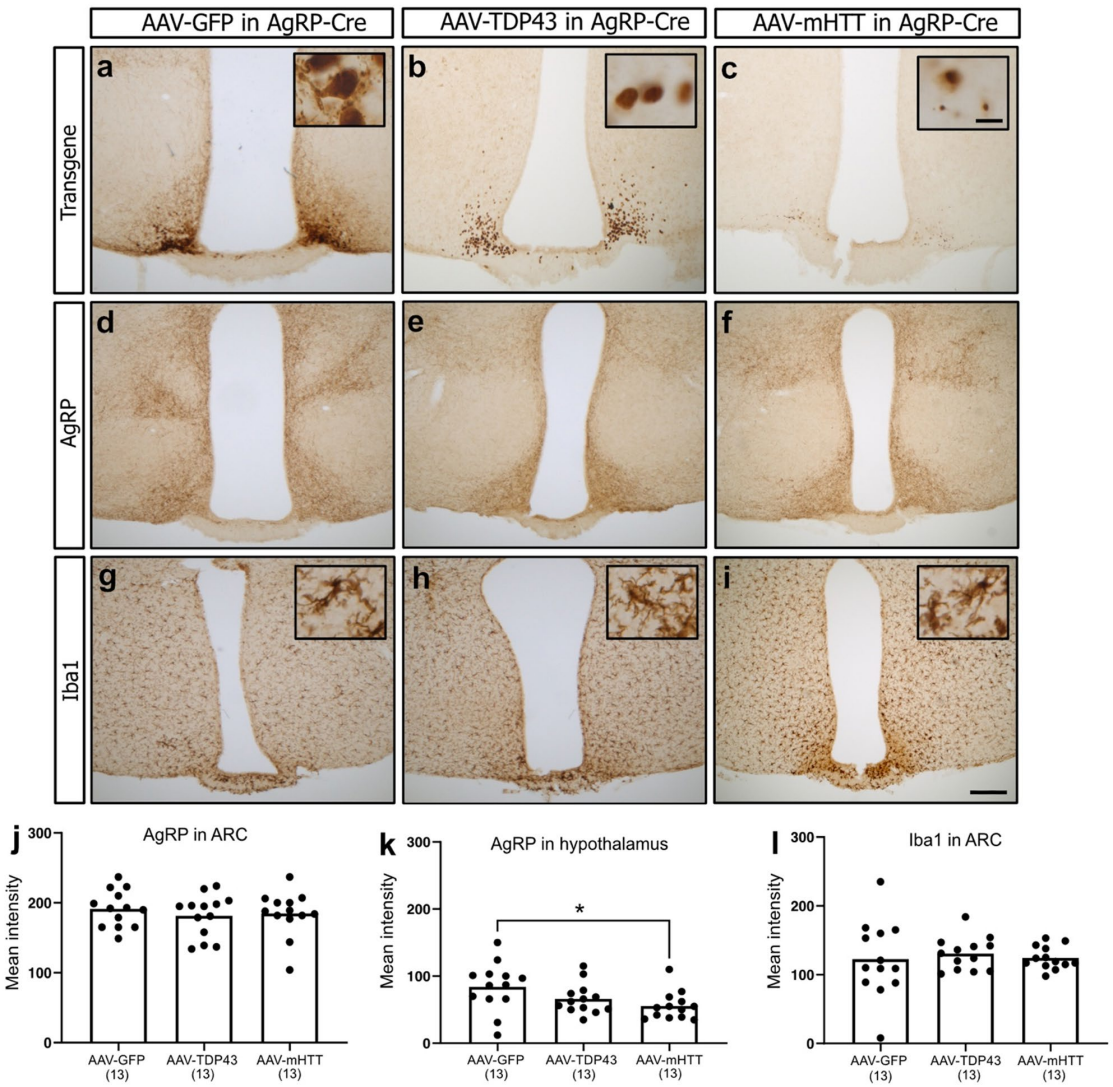
Neuropathological examination shows overexpression of mHTT but not TDP-43 significantly reduces total hypothalamic AgRP fibre expression with no effect of overexpression of TDP-43 or mHTT on the intensity of AgRP fibre and Iba1 expression in the ARC. Representative images show targeted expression of GFP, TDP-43 and mHTT in the ARC 21-weeks after bilateral stereotactic injections of AAV-GFP, AAV-TDP43 and AAV-mHTT in the hypothalamus of AgRP-cre mice (**a**–**c**). There was no difference between the groups in intensity of AgRP immunopositive fibres in the ARC (**d**–**f**, **j**–**k**). Reduced AgRP intensity in the hypothalamus (**k**) was observed in AAV-mHTT injected brains compared to AAV-GFP injected brains. There was no difference between the group in Iba1 intensity in the ARC (**j**–**l**). Data was analyzed by two-way ANOVA for the influence of sex and vector, with a subsequent one-way ANOVA if the effect of sex or vector was established, followed by Tukey’s multiple-comparison test of significant effects. *=0.035. The number of samples in each group is indicated in parentheses. Scale bar in c insert = 5 μm. Scale bar in i = 200 μm

We performed immunohistochemistry on AAV-GFP (*n* = 13), AAV-TDP43 (*n* = 13) and AAV-mHTT (*n* = 13) injected brains at 21-weeks post injection (Fig. 4a–i). Expression of the transgenes was localised to the ARC in all three groups (Fig. 4a–c), with a prominent appearance of mHTT inclusions at this time-point (Fig. 4c). Two-way ANOVA revealed that AgRP fibres in the hypothalamus were significantly reduced dependent on vector type (Fig. 4. k, *p =* 0.039), but there was no sex-dependent effect (*p* = 0.392). One-way ANOVA revealed a significant reduction on the intensity of the immunoreactivity of AgRP fibres in the hypothalamus after AAV-mHTT injection compared to AAV-GFP (*p =* 0.032). Overexpression of mHTT lead to a 34% reduction of AgRP fibres in the entire hypothalamus relative to overexpression of GFP. There was no effect of vector type (*p* = 0.690) or sex (*p* = 0.834) on AgRP fibers only in ARC (Fig. 4j).

#### No effect of TDP-43 and mHTT overexpression on neuroinflammation

A number of studies have indicated that microglia are activated in response to TDP-43 aggregation in clinical tissue and mouse models of ALS [69–71] Similarly, microglia have been found to be activated in the brains of persons with HD including in the hypothalamus [72–74]. We therefore investigated the effect of TDP-43 and mHTT overexpression in the ARC using immunohistochemistry for Iba1. However, we detected no apparent differences in the morphology of Iba1 immunoreactive microglia in the ARC between the different groups (Fig. 4g-i). We also assessed Iba1 intensity in the ARC, but a two-way ANOVA revealed no vector- (*p* = 0.577) or sex-dependent effect (*p* = 0.065) at 21-weeks post-injection (Fig. 4l). Hence, overexpression of TDP-43 and mHTT in the ARC does not appear to differentially activate microglia compared to overexpression of GFP.

### Analyses of TDP-43 and mHTT overexpression in the ARC of AgRP-Cre mice

**Fig. 5:**
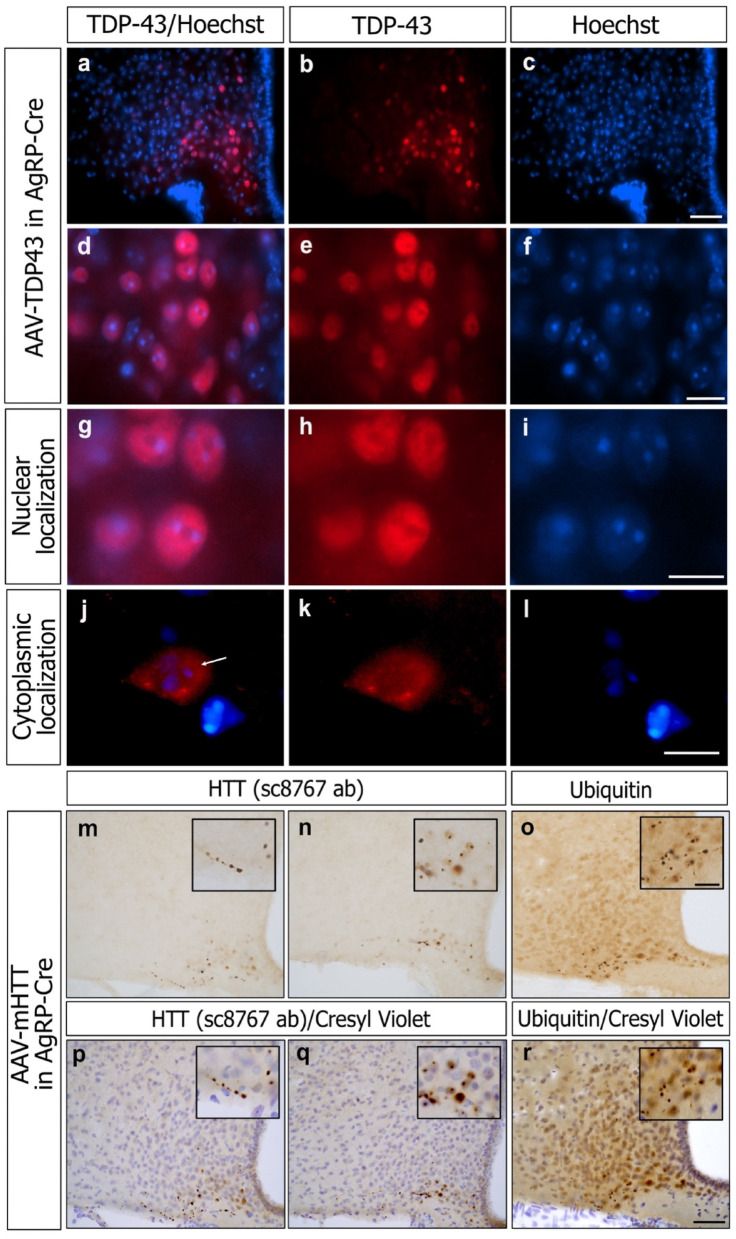
Analyses of TDP-43 localization and mHTT inclusions in AgRP-cre mice. Immunofluorescence of TDP-43 shows mainly localization of TDP-43 in the nucleus of AgRP neurons in the ARC after AAV-vector mediated overexpression (**a**–**i**). Mislocalization of TDP-43 from the nucleus to the cytoplasm was observed only in a few neurons (arrow,** j**–**l**). mHTT inclusions were detected using the sc8767 HTT antibody both intracellularly and in projections after overexpression of mHTT in the AgRP-cre mice 21-weeks-post-injection (**m**, **n**, **p**, **q**). Ubiquitin-immunopositive inclusions were detected intracellularly and in projections in AgRP-cre mice overexpressing mHTT (**o**,** r**) but not TDP-43 (data not shown). Scalebar in c, *r* = 50 μm. Scalebar in **f**, **i**, **l**, **o** insert = 10 μm.

Pathogenic TDP-43 is known to undergo nuclear-to-cytoplasmic mislocalization and form both nuclear and cytoplasmic inclusions in patients with FTD/ALS [75, 76]. To investigate the molecular effects following TDP-43 overexpression, we performed immunofluorescent analysis of brains injected with AAV-TDP43 in the ARC at 21-weeks post injection with an antibody against TDP-43 and counterstained with Hoechst for nuclear localization (*n* = 3) (Fig. 5a–i). TDP-43 was detected predominantly in the nucleus (Fig. 5g–i) but was also present in the cytoplasm of 2–4% of TDP-43 overexpressing cells (Fig. 5j–l).

Immunohistochemical staining of AAV-mHTT injected brains with the sc8767 antibody (with epitope mapping near the N-terminus of HTT; [38]) revealed mHTT inclusion formation intracellularly (Fig. 5n, q), but also what appeared to be within projections (Fig. 5m, p), which is commonly detected in HD and described as dystrophic neurites [5, 6].

We also performed immunohistochemistry with an antibody against the protein Sequestesome 1 (p62), that is involved in clearing protein aggregates through autophagy and is commonly used to label protein inclusions in several neurodegenerative disorders including FTD/ALS and HD [77–79]. We processed brains injected with AAV-GFP (*n* = 2), AAV-TDP43 (*n* = 2) and AAV-mHTT (*n* = 2) using an antibody against p62 in order to examine whether overexpression of TDP-43 and mHTT would lead to p62-immunoreactive inclusions in the ARC. However, our analyses showed no evidence of increased p62 immunoreactivity or p62 inclusion formation at 21-weeks post injection in either the AAV-TDP43 or AAV-mHTT compared to AAV-GFP control group (data not shown).

Finally, we investigated whether overexpression of TDP-43 and mHTT would lead to the formation of ubiquitinated inclusions in AgRP neurons. Ubiquitin, a protein tagged to cells undergoing degradation and autophagy, has been shown to be present in neuronal inclusions in FTD/ALS and HD [75, 80, 81]. We immunohistochemically processed AAV-TDP43 (*n* = 3) and AAV-mHTT (*n* = 3), as well as AAV-GFP (*n* = 3) injected brains 21-weeks post-injection. Our analyses showed formation of ubiquitin-immunopositive inclusions in AgRP cells following AAV-mHTT injection (Fig. 5o, r),

however no evidence for formation of ubiquitin-immunopositive inclusions in AAV-TDP43 or AAV-GFP injected brains (data not shown).

## Discussion

Pathology in the hypothalamus in the spectrum of FTD/ALS and HD may be linked to the development of early non-motor features with altered metabolism and psychiatric symptoms [24, 25, 32, 82]. Recent studies using neuroimaging and post-mortem neuropathological analyses of persons with these disorders have identified a number of changes in the hypothalamus [26–31, 83–85]. These changes also include associations between hypothalamic atrophy and reduced BMI in ALS as well as reduced oxytocin levels in the cerebrospinal fluid and social cognitive deficits in HD [28, 82, 86]. However, clinical studies are limited by difficulties in establishing causal structure-to-function relationships. AAV-vectors targeting transgenes to specific neurocircuitries provide opportunities to investigate casual effects from overexpression of proteins on the development of pathology and disease-related phenotypes. Here we used flex-switch AAV vectors to investigate the effects of wild-type TDP-43 and mHTT in AgRP neurons in the ARC of the hypothalamus in mice. Interestingly, overexpression of mHTT led to mHTT and ubiquitin-immunopositive inclusion formation as well as reduced AgRP-fiber intensity in the hypothalamus. We also observed an increase in food intake in female mice following mHTT overexpression. In contrast, overexpression of TDP-43 did not lead to ubiquitin- immunopositive inclusions or any altered phenotypes. Taken together, AgRP neurons in the ARC of the mouse hypothalamus do not appear sensitive to the effects of overexpression of wild-type TDP-43 at the time-point studied here whereas they display some sensitivity to the effects of mHTT overexpression in the hypothalamus within the same timeframe of 21 weeks post-injection.

We have recently shown that overexpression of TDP-43 in the entire hypothalamus leads to metabolic dysregulation with increased body weight and increased body fat mass in mice [34]. In this model, we found loss of hypocretin-, MCH- and oxytocin-expressing neurons following pan-hypothalamic overexpression of TDP-43 in neurons. Overexpression of TDP-43 was achieved using stereotactic injections of AAV-vectors expressing wild-type TDP-43 under the neuronal promoter synapsin in wild-type mice [34]. Changes in body weight were detected as early as 2 weeks-post-injection, altered nesting ability was detected at 8 weeks post-injection and neuropathology was confirmed at 10 weeks post-injection in this model. In the present study, we wanted to test the specific effects of overexpression of TDP-43 on AgRP-expressing neurons in the ARC. AgRP neurons are master regulators of energy homeostasis, promoting appetite and reducing energy expenditure [49, 87–89]. We found that the overexpression of TDP-43 in AgRP neurons did not affect body weight gain or food-intake in either male or female mice. Hence, other hypothalamic neuronal populations are more likely to be involved in the effects on metabolism resulting from TDP-43 overexpression in the hypothalamus.

Our previous work has also shown that overexpression of mHTT in the hypothalamus leads to metabolic dysregulation with increased body weight in mice [38]. However, in a clinical setting, persons with HD show significant weight loss despite unchanged food intake, or interestingly enough, despite being on a high caloric and fat diet [14, 26, 90]. To investigate whether overexpression of mHTT in AgRP neurons would lead to metabolic dysregulation, we overexpressed the same fragment of mHTT as used in previous studies in mice [38]. Here, we found no change in body weight in either male or female mice. However, AAV-mHTT injected female mice consumed more food per day compared to AAV-TDP43 and AAV-GFP injected females. These results align with our previous work showing that overexpression of mHTT leads to increased food intake in female mice [38, 55]. As we observed an increase in food intake with an unchanged body weight in female mice, one possible consideration is the sex-dimorphic traits in metabolic regulation [91, 92]. There are no clear sex differences for the disease manifestations in HD but it has not been studied in detail whether there may be specific changes in appetite or energy metabolism in women with HD. Interestingly, several studies have shown that female mice are resistant to the obesifying effects of a high fat diet compared to male mice [92–94], which may provide an understanding for the observation that female mice consumed more food whilst maintaining their body weight. To better understand the disparity between food intake and body weight found in this study, future studies could include an assessment of energy expenditure alongside food intake. Additionally, AgRP neurons are known to be stimulated by hunger signals, for example, fasting [95–97]. The activity of fasting induces an increase in the firing potential of AgRP neurons, therefore triggering a food seeking behaviour [98, 99]. As our animals had *ad libitum* access to standard chow throughout the duration of the study and there were no fasting periods, there was no great strive for seeking out food. To further assess the effect of mHTT on AgRP neurons, future studies could include analyses of fasting-induced food intake.

Despite minor effects on metabolism and no effects on behavior, overexpression of mHTT 21-weeks post injection significantly reduced the intensity of the immunoreactivity of AgRP fibres in the entire hypothalamus. Interestingly, we also detected mHTT inclusions in neuronal projections in the ARC. This is in line with previous work showing deficits in intracellular trafficking and axonal pathology including formation of mHTT inclusions in neuronal axons in models of HD [100–106]. This suggests that axonal mHTT inclusions may be associated with toxic effects on AgRP fibres.

Psychiatric features including anxiety and apathy, i.e. the lack of goal-directed activity and motivation, are common in HD and FTD/ALS [66, 107–111]. We therefore investigated the effect of TDP-43 and mHTT overexpression on apathy-like behaviour in the form of nest construction as well as on anxiety-like behavior in the EPM. However, mice injected with AAV-TDP43 or AAV-mHTT displayed no differences compared to mice injected with the control vector AAV-GFP. Hence, overexpression of TDP-43 or mHTT in the ARC does not appear to lead to altered apathy- and anxiety-like behavior in mice.

Compared to previous studies overexpressing TDP-43 and mHTT in the entire hypothalamus, we only detect modest metabolic and neuropathological (mHTT) or no (TDP-43) effects after overexpression of these proteins in AgRP neurons in the ARC. Although AgRP neurons modulate metabolic activity, they interact with other neuronal populations in the ARC to provide different functions. Neuropeptide Y (NPY) is abundantly expressed within the central nervous system (CNS) and resides mainly in the ARC. In fact, NPY-expressing neurons have been shown to be increased in post-mortem human HD brains compared to controls [112]. Additionally, NPY-expressing striatal neurons are spared in the R6/2 mouse model of HD, and interestingly enough, nasal administration of NPY leads to a significant reduction in the percentage of mHTT inclusion formation in the motor cortex and striatum of R6/2 mice [113–115]. At end-stage disease in the SOD1 mouse model of familial ALS, NPY populations significantly increased by 30% [116] indicating a protective role of NPY in neurodegeneration. Interestingly, previous studies have shown that while not all NPY neurons co-express AgRP [47, 117], all AgRP neurons co-express NPY [88, 118]. Although we did not investigate this relationship in our AgRP-Cre brains, the coexpression of NPY in AgRP neurons may lead to a resilience of AgRP neurons to the otherwise toxic effects of disease-related proteins and explain the lack of metabolic and behavioral phenotypes in our study.

In this study, we have used flex-switch AAV vectors to test whether overexpression of TDP-43 and mHTT specifically in AgRP neurons would lead to neuropathology and effects on metabolism and behavior. Although we achieve selective targeting to AgRP neurons, these proteins are overexpressed which are not the case in the spectrum of FTD/ALS and HD. Despite this discrepancy between the mouse model and the human disease, mHTT inclusions were formed after overexpression of mHTT both intracellularly as well as in projections, which is similar to what has been reported in neuropathological studies of clinical HD [5–7]. On the other hand, most TDP-43 protein expressed from the flex-switch AAV-vector remained in the nucleus and only approximately 2–4% of the transfected cells displayed cytoplasmic translocation of TDP-43. This is different to typical pathology in TDP-43 proteinopathies where nuclear TDP-43 is depleted leading to loss of nuclear TDP-43 function, and TDP-43 is translocated to the cytoplasm leading to gain-of-function effects [2, 11, 75, 76] Hence, the low abundance of cytoplasmic translocation of TDP-43 is a limitation with the present study and it is possible that other results would have been detected if more AgRP neurons would have displayed cytoplasmic translocation of TDP-43.

An aspect that would be highly relevant to study would be the potential crosstalk between TDP-43 and mHTT in generating neuropathology. Clinical studies of cortical and striatal postmortem tissues have shown TDP-43 aggregation in cases with HD as well as mHTT inclusions in a few cases with both intermediate (27–35 CAG repeats) or reduced penetrance (26–39 CAG repeats) HTT alleles and motor neuron disease, suggesting a potential overlap in the neuropathology of these diseases [12, 13, 119]. It is not yet known whether any such overlap exists in human postmortem tissue from the hypothalamus in FTD/ALS and HD cases. Future experimental studies with coexpression of these proteins may yield further mechanistic insight into the development of pathology in these disorders.

In conclusion, this study shows that overexpression of mHTT in the ARC has a sex-specific effect on food intake and leads to formation of mHTT inclusions as well as a reduction in AgRP fibres compared to overexpression of GFP, suggesting that the presence of mHTT inclusions in fibres may lead to axonal pathology. In contrast, overexpression of wild-type TDP-43 in the ARC does not lead to pathology or to the development of metabolic and psychiatric-like features in mice. Taken together, overexpression of wild-type TDP-43 in AgRP neurons does not lead to early toxicity whereas overexpression of mHTT results in mHTT inclusions and AgRP fibre toxicity in mice. It is therefore possible that other neuronal populations in the hypothalamus are more vulnerable to the effects of the disease-causing proteins TDP-43 and mHTT as has been reported in the context of FTD/ALS and HD [25, 27, 29, 30, 32].

## Supplementary Information

Below is the link to the electronic supplementary material.

Supplementary Material 1

## Data Availability

The datasets analysed during the current study are available from the corresponding author on reasonable request.

## Abbreviations

AAV vector: Adeno associated viral vector
AAV-GFP: AAV-vector expressing GFP
AAV-TDP43: AAV-vector expressing TDP-43
AAV-mHTT: AAV-vector expressing huntingtin with 78 polyglutamines
AgRP: Agouti-related protein
AgRP-Cre: Agouti-related protein Cre-recombinase mouse model
ALS: Amyotrophic lateral sclerosis
ANOVA: Analysis of Variance
ARC: Arcuate nucleus of the hypothalamus
CV: Cresyl violet
DAB: 3,3’-Diaminobenzidine
DPX: Dibutylphthalate Polystyrene Xylene
EPM: Elevated plus maze
EYFP: Enhanced yellow fluorescent protein
GFP: Green fluorescent protein
HD: Huntington’s disease
HTT: Huntingtin protein
Iba1: Ionized calcium binding adaptor molecule 1
IRES: Internal ribosome entry site
mHTT: Mutant huntingtin protein
NPY: Neuropeptide Y
OF: Open field test
p62: Protein Sequestesome 1
SD: Standard deviation
TDP-43: TAR DNA-binding protein of 43 kDa
