# Supplementary material for: Differential effects of overexpression of mutant huntingtin and TDP-43 in agouti-related protein neurons in the arcuate nucleus of the hypothalamus in mice
Source: Acta Neuropathol Commun. 2025 Dec 7;13:253. doi: 10.1186/s40478-025-02201-x (PMC12706952; doi:10.1186/s40478-025-02201-x)
Supplement: Supplementary file 1 — Supplementary Material 1 [file 40478_2025_2201_MOESM1_ESM.docx]

**Supplementary information S1**

**Differential effects of overexpression of mutant huntingtin and TDP-43 in agouti-related protein neurons in the arcuate nucleus of the hypothalamus in mice**

Jennifer Oraha^1^, Ronja Wagner^1^, Sofia Bergh^1^, Nicola J Lee^2,3^,

Deniz Kirik^4^, and Åsa Petersén^1, 5^

^1^Translational Neuroendocrine Unit, Experimental Medical Sciences, Medical Faculty, Lund University, Sweden

^2^Charles Perkins Centre, University of Sydney, NSW, Australia

^3^School of Life and Environmental Sciences, Faculty of Science, University of Sydney, NSW, Australia

^4^Brain repair and Imaging in Neural Systems, Experimental Medical Sciences, Medical Faculty, Lund University, Sweden

^5^Department of Psychiatry, Skåne University Hospital, Lund, Sweden

**Corresponding author:**

Correspondence should be addressed to Åsa Petersén (email: asa.petersen@med.lu.se), Translational Neuroendocrine Research Unit, Department of Experimental Medical Science, Lund University, BMC D11, 221 84 Lund, Sweden.

**Appendix 1: Supplementary Statistical Results**

**Figure 1**

**(Fig 1. n , o )** % body weight change at 10-weeks post-injection

Two-way ANOVA

Sex: F= 9.148, df= 1 p=0.006

Vector: F=0.542, df= 2 p=0.589

Sex*Vector: F=0.287, df= 2 p=0.753

One-way ANOVA

Sex(MALE): F=1.015, df=2, p=0.388

| **Multiple Comparisons** | | | |
| --- | --- | --- | --- |
| Dependent Variable: % body weight change | | | |
| *Tukey’s post hoc* | | | |
| Vector | Vector | p-value |  |
|  |  |  |  |
| AAV-GFP | AAV-TDP43 | 0.799 |  |
|  | AAV-mHTT | 0.692 |  |
| AAV-TDP43 | AAV-GFP | 0.799 |  |
|  | AAV-mHTT | 0.369 |  |
| AAV-mHTT | AAV-GFP | 0.692 |  |
|  | AAV-TDP43 | 0.369 |  |
|  | | | |

One-way ANOVA

Sex(FEMALE): F=0.145, df=2, p=0.867

| **Multiple Comparisons** | | | |
| --- | --- | --- | --- |
| Dependent Variable: % body weight change | | | |
| *Tukey’s post hoc* | | | |
| Vector | Vector | p-value |  |
|  |  |  |  |
| AAV-GFP | AAV-TDP43 | 0.856 |  |
|  | AAV-mHTT | 0.946 |  |
| AAV-TDP43 | AAV-GFP | 0.856 |  |
|  | AAV-mHTT | 0.955 |  |
| AAV-mHTT | AAV-GFP | 0.946 |  |
|  | AAV-TDP43 | 0.955 |  |
|  | | | |
|  | | | |

|  |
| --- |

**Figure 2**

**(Fig 2 b, c):** body weight (g) over 21 weeks

**Two-way ANOVA**

| Source | Dependent Variable | df | F | p-value |
| --- | --- | --- | --- | --- |
| Sex | w17 | 1 | 127.010 | <.001 |
|  | w 22 | 1 | 152.934 | <.001 |
|  | w 26 | 1 | 124.955 | <.001 |
|  | w 30 | 1 | 109.559 | <.001 |
|  | w 34 | 1 | 98.479 | <.001 |
|  | w 38 | 1 | 62.535 | <.001 |
| Vector | w17 | 2 | 1.314 | .282 |
|  | w 22 | 2 | .064 | .938 |
|  | w 26 | 2 | .299 | .744 |
|  | w 30 | 2 | .320 | .728 |
|  | w 34 | 2 | .557 | .578 |
|  | w 38 | 2 | .182 | .834 |
| Sex* Vector | w17 | 2 | .918 | .409 |
|  | w 22 | 2 | .716 | .496 |
|  | w 26 | 2 | .460 | .635 |
|  | w 30 | 2 | .080 | .923 |
|  | w 34 | 2 | .109 | .897 |
|  | w 38 | 2 | .138 | .871 |

One-way ANOVA

sex(MALE)

0weeks: F=1.331, df=2, p=0.294

4weeks: F=0.680, df=2, p=0.680

8weeks: F=0.376, df=2, p=0.693

12weeks: F=0.241, df=2, p=0.789

16weeks: F=0.144, df=2, p=0.867

21weeks: F=0.300, df=2, p=0.745

| **Multiple Comparisons** | | | |
| --- | --- | --- | --- |
| Dependent Variable: body weight 0weeks | | | |
| *Tukey’s post hoc* | | | |
| Vector | Vector | p-value |  |
|  |  |  |  |
| AAV-GFP | AAV-TDP43 | 0.556 |  |
|  | AAV-mHTT | 0.295 |  |
| AAV-TDP43 | AAV-GFP | 0.556 |  |
|  | AAV-mHTT | 0.894 |  |
| AAV-mHTT | AAV-GFP | 0.295 |  |
|  | AAV-TDP43 | 0.894 |  |
|  | | | |
| **Multiple Comparisons** | | | |
| Dependent Variable: body weight 4weeks | | | |
| *Tukey’s post hoc* | | | |
| Vector | Vector | p-value |  |
|  |  |  |  |
| AAV-GFP | AAV-TDP43 | 0.991 |  |
|  | AAV-mHTT | 0.672 |  |
| AAV-TDP43 | AAV-GFP | 0.991 |  |
|  | AAV-mHTT | 0.788 |  |
| AAV-mHTT | AAV-GFP | 0.672 |  |
|  | AAV-TDP43 | 0.788 |  |
|  | | | |

| **Multiple Comparisons** | | | |
| --- | --- | --- | --- |
| Dependent Variable: body weight 8weeks | | | |
| *Tukey’s post hoc* | | | |
| Vector | Vector | p-value |  |
|  |  |  |  |
| AAV-GFP | AAV-TDP43 | 0.959 |  |
|  | AAV-mHTT | 0.670 |  |
| AAV-TDP43 | AAV-GFP | 0.959 |  |
|  | AAV-mHTT | 0.857 |  |
| AAV-mHTT | AAV-GFP | 0.670 |  |
|  | AAV-TDP43 | 0.857 |  |
|  | | | |

| **Multiple Comparisons** | | | |
| --- | --- | --- | --- |
| Dependent Variable: body weight 12weeks | | | |
| *Tukey’s post hoc* | | | |
| Vector | Vector | p-value |  |
|  |  |  |  |
| AAV-GFP | AAV-TDP43 | 0.848 |  |
|  | AAV-mHTT | 0.821 |  |
| AAV-TDP43 | AAV-GFP | 0.848 |  |
|  | AAV-mHTT | 0.999 |  |
| AAV-mHTT | AAV-GFP | 0.821 |  |
|  | AAV-TDP43 | 0.999 |  |
|  | | | |

| **Multiple Comparisons** | | | |
| --- | --- | --- | --- |
| Dependent Variable: body weight 16weeks | | | |
| *Tukey’s post hoc* | | | |
| Vector | Vector | p-value |  |
|  |  |  |  |
| AAV-GFP | AAV-TDP43 | 0.929 |  |
|  | AAV-mHTT | 0.871 |  |
| AAV-TDP43 | AAV-GFP | 0.929 |  |
|  | AAV-mHTT | 0.992 |  |
| AAV-mHTT | AAV-GFP | 0.871 |  |
|  | AAV-TDP43 | 0.992 |  |
|  | | | |

| **Multiple Comparisons** | | | |
| --- | --- | --- | --- |
| Dependent Variable: body weight 21weeks | | | |
| *Tukey’s post hoc* | | | |
| Vector | Vector | p-value |  |
|  |  |  |  |
| AAV-GFP | AAV-TDP43 | 0.759 |  |
|  | AAV-mHTT | 0.846 |  |
| AAV-TDP43 | AAV-GFP | 0.759 |  |
|  | AAV-mHTT | 0.989 |  |
| AAV-mHTT | AAV-GFP | 0.846 |  |
|  | AAV-TDP43 | 0.989 |  |
|  | | | |

One-way ANOVA

sex(FEMALE)

0weeks: F=1.129, df=2, p=0.345

4weeks: F=0.424, df=2, p=0.661

8weeks: F=0.411, df=2, p=0.669

12weeks: F=0.165, df=2, p=0.849

16weeks: F=0.674, df=2, p=0.522

21weeks: F=0.003, df=2, p=0.997

| **Multiple Comparisons** | | | |
| --- | --- | --- | --- |
| Dependent Variable: body weight 0weeks | | | |
| *Tukey’s post hoc* | | | |
| Vector | Vector | p-value |  |
|  |  |  |  |
| AAV-GFP | AAV-TDP43 | 0.487 |  |
|  | AAV-mHTT | 1.000 |  |
| AAV-TDP43 | AAV-GFP | 0.487 |  |
|  | AAV-mHTT | 0.381 |  |
| AAV-mHTT | AAV-GFP | 1.000 |  |
|  | AAV-TDP43 | 0.381 |  |
|  | | | |

| **Multiple Comparisons** | | | |
| --- | --- | --- | --- |
| Dependent Variable: body weight 4weeks | | | |
| *Tukey’s post hoc* | | | |
| Vector | Vector | p-value |  |
|  |  |  |  |
| AAV-GFP | AAV-TDP43 | 0.948 |  |
|  | AAV-mHTT | 0.876 |  |
| AAV-TDP43 | AAV-GFP | 0.948 |  |
|  | AAV-mHTT | 0.638 |  |
| AAV-mHTT | AAV-GFP | 0.876 |  |
|  | AAV-TDP43 | 0.638 |  |
|  | | | |
| **Multiple Comparisons** | | | |
| Dependent Variable: body weight 8weeks | | | |
| *Tukey’s post hoc* | | | |
| Vector | Vector | p-value |  |
|  |  |  |  |
| AAV-GFP | AAV-TDP43 | 0.763 |  |
|  | AAV-mHTT | 1.000 |  |
| AAV-TDP43 | AAV-GFP | 0.763 |  |
|  | AAV-mHTT | 0.695 |  |
| AAV-mHTT | AAV-GFP | 1.000 |  |
|  | AAV-TDP43 | 0.695 |  |
|  | | | |

| **Multiple Comparisons** | | | |
| --- | --- | --- | --- |
| Dependent Variable: body weight 12weeks | | | |
| *Tukey’s post hoc* | | | |
| Vector | Vector | p-value |  |
|  |  |  |  |
| AAV-GFP | AAV-TDP43 | 0.857 |  |
|  | AAV-mHTT | 0.987 |  |
| AAV-TDP43 | AAV-GFP | 0.857 |  |
|  | AAV-mHTT | 0.903 |  |
| AAV-mHTT | AAV-GFP | 0.987 |  |
|  | AAV-TDP43 | 0.903 |  |
|  | | | |

| **Multiple Comparisons** | | | |
| --- | --- | --- | --- |
| Dependent Variable: body weight 16weeks | | | |
| *Tukey’s post hoc* | | | |
| Vector | Vector | p-value |  |
|  |  |  |  |
| AAV-GFP | AAV-TDP43 | 0.492 |  |
|  | AAV-mHTT | 0.791 |  |
| AAV-TDP43 | AAV-GFP | 0.492 |  |
|  | AAV-mHTT | 0.836 |  |
| AAV-mHTT | AAV-GFP | 0.791 |  |
|  | AAV-TDP43 | 0.836 |  |
|  | | | |

| **Multiple Comparisons** | | | |
| --- | --- | --- | --- |
| Dependent Variable: body weight 21weeks | | | |
| *Tukey’s post hoc* | | | |
| Vector | Vector | p-value |  |
|  |  |  |  |
| AAV-GFP | AAV-TDP43 | 0.999 |  |
|  | AAV-mHTT | 0.997 |  |
| AAV-TDP43 | AAV-GFP | 0.999 |  |
|  | AAV-mHTT | 0.999 |  |
| AAV-mHTT | AAV-GFP | 0.997 |  |
|  | AAV-TDP43 | 0.999 |  |
|  | | | |

**Figure 2**

**(Fig 2 d, e):** % body weight change at 21-weeks post-injection

Two-way ANOVA

Sex: F= 8.182, df= 1 p=0.007

Vector: F=1.114, df= 2 p=0.340

Sex*Vector: F=0.524, df= 2 p=0.597

One-way ANOVA

sex (FEMALE): F=0.361, df=2, p=0.702

| **Multiple Comparisons** | | | |
| --- | --- | --- | --- |
| Dependent Variable: % body weight change | | | |
| *Tukey’s post hoc* | | | |
| Vector | Vector | p-value |  |
|  |  |  |  |
| AAV-GFP | AAV-TDP43 | 0.770 |  |
|  | AAV-mHTT | 0.999 |  |
| AAV-TDP43 | AAV-GFP | 0.770 |  |
|  | AAV-mHTT | 0.741 |  |
| AAV-mHTT | AAV-GFP | 0.999 |  |
|  | AAV-TDP43 | 0.741 |  |
|  | | | |

One-way ANOVA

SEX (MALE): F=1.376, df=2, p=0.283

| **Multiple Comparisons** | | | |
| --- | --- | --- | --- |
| Dependent Variable: % body weight change | | | |
| *Tukey’s post hoc* | | | |
| Vector | Vector | p-value |  |
|  |  |  |  |
| AAV-GFP | AAV-TDP43 | 0.411 |  |
|  | AAV-mHTT | 0.345 |  |
| AAV-TDP43 | AAV-GFP | 0.411 |  |
|  | AAV-mHTT | 0.992 |  |
| AAV-mHTT | AAV-GFP | 0.345 |  |
|  | AAV-TDP43 | 0.992 |  |
|  | | | |

**Figure 2**

**(Fig 2 f, g):** Daily food intake per mouse 16-weeks post-injection

Two-way ANOVA

Sex: F= 22.345 , df= 1, p<0.001

Vector: F= 2.898, df= 2 p= 0.069

Sex*Vector: F= 2.135, df= 2 p= 0.134

One-way ANOVA

Sex(FEMALE): F= 5.867, df=2, p=0.012

| **Multiple Comparisons** | | | |
| --- | --- | --- | --- |
| Dependent Variable: Daily food intake | | | |
| *Tukey’s post hoc* | | | |
| Vector | Vector | p-value |  |
|  |  |  |  |
| AAV-GFP | AAV-TDP43 | 0.592 |  |
|  | AAV-mHTT | *0.013 |  |
| AAV-TDP43 | AAV-GFP | 0.592 |  |
|  | AAV-mHTT | *0.049 |  |
| AAV-mHTT | AAV-GFP | *0.013 |  |
|  | AAV-TDP43 | *0.049 |  |
| *. The mean difference is significant at the 0.05 level. | | | |

One-way ANOVA

Sex(MALE): F=0.247, df=2, p=0.784

| **Multiple Comparisons** | | | |
| --- | --- | --- | --- |
| Dependent Variable: Daily food intake | | | |
| *Tukey’s post hoc* | | | |
| Vector | Vector | p-value |  |
|  |  |  |  |
| AAV-GFP | AAV-TDP43 | 0.813 |  |
|  | AAV-mHTT | 0.999 |  |
| AAV-TDP43 | AAV-GFP | 0.813 |  |
|  | AAV-mHTT | 0.801 |  |
| AAV-mHTT | AAV-GFP | 0.999 |  |
|  | AAV-TDP43 | 0.801 |  |
|  | | | |
|  | | | |

**Figure 2**

**(Fig 2 g, h):** Normalised food intake

Two-way ANOVA

Sex: F= 22.345, df=1 p<0.001

Vector: F=0.006 df=2 p=0.003

Sex*Vector: F=03.022, df=2 p=0.062

One-Way ANOVA

Sex(FEMALE): F=6.769, df=2, p=0.007

| **Multiple Comparisons** | | | |
| --- | --- | --- | --- |
| Dependent Variable: Normalised food intake | | | |
| *Tukey’s post hoc* | | | |
| Vector | Vector | p-value |  |
|  |  |  |  |
| AAV-GFP | AAV-TDP43 | 0.292 |  |
|  | AAV-mHTT | *0.006 |  |
| AAV-TDP43 | AAV-GFP | 0.292 |  |
|  | AAV-mHTT | 0.068 |  |
| AAV-mHTT | AAV-GFP | *0.006 |  |
|  | AAV-TDP43 | 0.068 |  |
| *. The mean difference is significant at the 0.05 level | | | |
|  | | | |

One-Way ANOVA

Sex(MALE): F=0.844, df=2, p=0.447

| **Multiple Comparisons** | | | |
| --- | --- | --- | --- |
| Dependent Variable: Normalised food intake | | | |
| *Tukey’s post hoc* | | | |
| Vector | Vector | p-value |  |
|  |  |  |  |
| AAV-GFP | AAV-TDP43 | 0.978 |  |
|  | AAV-mHTT | 0.540 |  |
| AAV-TDP43 | AAV-GFP | 0.978 |  |
|  | AAV-mHTT | 0.499 |  |
| AAV-mHTT | AAV-GFP | 0.540 |  |
|  | AAV-TDP43 | 0.499 |  |
|  | | | |

**Figure 3**

**(Fig 3 a, b):** Latency to fall in the rotarod at 18-weeks post-injection

Two-way ANOVA

Sex: F= 10.806, df=1 p=0.003

Vector: F=0.006 df=2 p=0.994

Sex*Vector: F=0.168, df=2 p=0.846

One-Way ANOVA

Sex(FEMALE): F=0.153, df=2, p=0.860

| **Multiple Comparisons** | | | |
| --- | --- | --- | --- |
| Dependent Variable: Latency to fall in Rotarod | | | |
| *Tukey’s post hoc* | | | |
| Vector | Vector | p-value |  |
|  |  |  |  |
| AAV-GFP | AAV-TDP43 | 0.884 |  |
|  | AAV-mHTT | 0.999 |  |
| AAV-TDP43 | AAV-GFP | 0.884 |  |
|  | AAV-mHTT | 0.894 |  |
| AAV-mHTT | AAV-GFP | 0.999 |  |
|  | AAV-TDP43 | 0.894 |  |
|  | | | |
|  | | | |

One-Way ANOVA

Sex(MALE): F=0.054, df=2, p=0.947

| **Multiple Comparisons**^a^ | | | |
| --- | --- | --- | --- |
| Dependent Variable: Latency to fall in Rotarod | | | |
| *Tukey’s post hoc* | | | |
| Vector | Vector | p-value |  |
|  |  |  |  |
| AAV-GFP | AAV-TDP43 | 0.970 |  |
|  | AAV-mHTT | 0.994 |  |
| AAV-TDP43 | AAV-GFP | 0.970 |  |
|  | AAV-mHTT | 0.944 |  |
| AAV-mHTT | AAV-GFP | 0.994 |  |
|  | AAV-TDP43 | 0.944 |  |
|  | | | |

**Figure 3**

**(Fig 3 c, d):** Total distance moved in Open Field at 17-weeks post-injection

Two-way ANOVA

Sex: F=9.795 , df=1 p=0.004

Vector: F=0.535 df=2 p=0.591

Sex*Vector: F=0.911, df=2 p=0.412

One-way ANOVA

Sex(FEMALE): F= 0.332, df=2, p=0.722

| **Multiple Comparisons** | | | |
| --- | --- | --- | --- |
| Dependent Variable: Total distance moved (m) | | | |
| *Tukey’s post hoc* | | | |
| Vector | Vector | p-value |  |
|  |  |  |  |
| AAV-GFP | AAV-TDP43 | 0.702 |  |
|  | AAV-mHTT | 0.917 |  |
| AAV-TDP43 | AAV-GFP | 0.702 |  |
|  | AAV-mHTT | 0.907 |  |
| AAV-mHTT | AAV-GFP | 0.917 |  |
|  | AAV-TDP43 | 0.907 |  |
|  | | | |
|  | | | |

One-way ANOVA

Sex(MALE): F= 1.133, df=2, p=0.344

| **Multiple Comparisons** | | | |
| --- | --- | --- | --- |
| Dependent Variable: Total distance moved (m) | | | |
| *Tukey’s post hoc* | | | |
| Vector | Vector | p-value |  |
|  |  |  |  |
| AAV-GFP | AAV-TDP43 | 0.893 |  |
|  | AAV-mHTT | 0.538 |  |
| AAV-TDP43 | AAV-GFP | 0.538 |  |
|  | AAV-mHTT | 0.339 |  |
| AAV-mHTT | AAV-GFP | 0.538 |  |
|  | AAV-TDP43 | 0.339 |  |

**Figure 3**

**(Fig 3 e, f):** Total distance moved in Elevated Plus Maze at 17-weeks post injection

Two-way ANOVA

Sex: F=6.473 , df=1, p=0.016

Vector: F=0.055 df=2 p=0.947

Sex*Vector: F=0.006, df=2 p=0.994

One-way ANOVA

Sex(FEMALE): F= 0.016, df=2, p=0.985

| **Multiple Comparisons** | | | |
| --- | --- | --- | --- |
| Dependent Variable: Total distance moved (m) | | | |
| *Tukey’s post hoc* | | | |
| Vector | Vector | p-value |  |
|  |  |  |  |
| AAV-GFP | AAV-TDP43 | 0.991 |  |
|  | AAV-mHTT | 1.000 |  |
| AAV-TDP43 | AAV-GFP | 0.991 |  |
|  | AAV-mHTT | 0.985 |  |
| AAV-mHTT | AAV-GFP | 1.000 |  |
|  | AAV-TDP43 | 0.985 |  |

One-way ANOVA

Sex(MALE): F= 0.047, df=2, p=0.954

| **Multiple Comparisons** | | | |
| --- | --- | --- | --- |
| Dependent Variable: Total distance moved (m) | | | |
| *Tukey’s post hoc* | | | |
| Vector | Vector | p-value |  |
|  |  |  |  |
| AAV-GFP | AAV-TDP43 | 0.986 |  |
|  | AAV-mHTT | 0.984 |  |
| AAV-TDP43 | AAV-GFP | 0.986 |  |
|  | AAV-mHTT | 0.950 |  |
| AAV-mHTT | AAV-GFP | 0.984 |  |
|  | AAV-TDP43 | 0.950 |  |

**Figure 3**

**(Fig 3 g):** %time spend in open arms in Elevated Plus Maze at 17-weeks post injection

Two-way ANOVA

Sex: F=0.133 , df=1 p=0.718

Vector: F=0.705 df=2 p=0.502

Sex*Vector: F=0.105, df=2 p=0.901

**Figure 3**

**(Fig 3 h):** Median nesting score day 2

| **Independent-Samples Kruskal-Wallis Test Summary: For vector** | |
| --- | --- |
| Total N | 39 |
| Test Statistic | 1.152^a^ |
| Degree Of Freedom | 2 |
| Asymptotic Sig.(2-sided test) | .562 |
| a. The test statistic is adjusted for ties. | |

| **Independent-Samples Kruskal-Wallis Test Summary: For Sex** | |
| --- | --- |
| Total N | 39 |
| Test Statistic | 6.564^a,b^ |
| Degree Of Freedom | 1 |
| Asymptotic Sig.(2-sided test) | 0.101 |
| a. The test statistic is adjusted for ties. | |
| b. Multiple comparisons are not performed because there are less than three test fields. | |

**(Fig 3 i):** Median nesting score day 4

| **Independent-Samples Kruskal-Wallis Test Summary: For sex** | |
| --- | --- |
| Total N | 39 |
| Test Statistic | .655^a,^ |
| Degree Of Freedom | 1 |
| Asymptotic Sig.(2-sided test) | .418 |
| a. The test statistic is adjusted for ties. | |
|  | |

| **Independent-Samples Kruskal-Wallis Test Summary: For vector** | |
| --- | --- |
| Total N | 39 |
| Test Statistic | 3.455^a^ |
| Degree Of Freedom | 2 |
| Asymptotic Sig.(2-sided test) | .178 |
| a. The test statistic is adjusted for ties. | |

**Figure 4**

**(Fig 4 j):** AgRP in ARC

Two-way ANOVA

Sex: F=0.045 , df=1, p=0.834

Vector: F=0.288, df=2, p=0.752

Sex*Vector: F=0.673, df=2 , p=0.517

One-way ANOVA

AgRP ARC fibres: F=0.853 , df=2, p=0.44

| **Multiple Comparisons** | | | |
| --- | --- | --- | --- |
| Dependent Variable: AgRP in ARC | | | |
| *Tukey’s post hoc* | | | |
| Vector | Vector | p-value |  |
|  |  |  |  |
| AAV-GFP | AAV-TDP43 | 0.515 |  |
|  | AAV-mHTT | 0.994 |  |
| AAV-TDP43 | AAV-GFP | 0.515 |  |
|  | AAV-mHTT | 0.547 |  |
| AAV-mHTT | AAV-GFP | 0.994 |  |
|  | AAV-TDP43 | 0.547 |  |
|  | | | |

**(Fig 4 k):** AgRP in hypothalamus

Two-way ANOVA

Sex: F=0.754 , df=1, p=0.392

Vector: F=2.638, df=2, p=0.039

Sex*Vector: F=0.061, df=2, p=0.941

One-way ANOVA

Treatment: F=3.563 , df=2, p=0.039

| **Multiple Comparisons** | | | |
| --- | --- | --- | --- |
| Dependent Variable: AgRP in hypothalamus | | | |
| *Tukey’s post hoc* | | | |
| Vector | Vector | p-value |  |
|  |  |  |  |
| AAV-GFP | AAV-TDP43 | 0.232 |  |
|  | AAV-mHTT | *0.032 |  |
| AAV-TDP43 | AAV-GFP | 0.232 |  |
|  | AAV-mHTT | 0.598 |  |
| AAV-mHTT | AAV-GFP | 0.032 |  |
|  | AAV-TDP43 | 0.598 |  |
| *The mean difference is significant at the 0.05 level | | | |
|  | | | |

**(Fig 4 l):** Iba1 in ARC

Two-way ANOVA

Sex: F=0.860 , df=1, p=0.060

Vector: F=0.559 df=2, p=0.577

Sex*Vector: F=0.860, df=2, p=0.432

One-way ANOVA

Treatment: F=0.165 , df=2, p=0.849

| **Multiple Comparisons** | | | |
| --- | --- | --- | --- |
| Dependent Variable: Iab1 in ARC | | | |
| *Tukey’s post hoc* | | | |
| Vector | Vector | p-value |  |
|  |  |  |  |
| AAV-GFP | AAV-TDP43 | 0.847 |  |
|  | AAV-mHTT | 0.990 |  |
| AAV-TDP43 | AAV-GFP | 0.847 |  |
|  | AAV-mHTT | 0.910 |  |
| AAV-mHTT | AAV-GFP | 0.990 |  |
|  | AAV-TDP43 | 0.910 |  |
|  | | | |
